# Experimental validation of a new technique for the assessment of posterior tibial translation (ABC angle) after posterior cruciate ligament rupture

**DOI:** 10.1186/s40634-021-00395-2

**Published:** 2021-09-03

**Authors:** M. Severyns, M.-E. Rollet, T. Vendeuvre, S. Pesenti, A. Benzakour, J.-L. Rouvillain

**Affiliations:** 1 Orthopaedics and Traumatology Department, CHU Martinique, F-97200 Fort-de-France, France; 2 Orthopaedics and Traumatology Department, CHU Poitiers, F-86021 Poitiers, France; 3 Paediatric Orthopaedics Department, CHU La Timone, F-13005 Marseilles, France; 4 Orthopaedics Department, Clinique de l’Archette, F-45160 Olivet, France

**Keywords:** Posterior translation, Posterior cruciate ligament, Angle measurement

## Abstract

**Background:**

The aim of this cadaver study was to evaluate an original technique for measuring posterior tibial translation based on an angle value instead of a distance value, with and without posterior stress application. It was hypothesized that an angle measurement of the posterior tibial translation would confirm the presence of a PCL tear with the knee flexed and completely extended.

**Method:**

Using fresh cadavers, a set of strict lateral views were taken by fluoroscopy with the knee at 0°, 45° and 90° flexion on the intact knee and after transecting the PCL. The primary endpoint was the change in the posterior translation measured using a new technique, the ABC angle. This measurement was compared to the conventional posterior translation distance measurement with and without a posterior stress placed on the knee.

**Results:**

Application of a posterior stress revealed clear changes in posterior translation after PCL transection with the knee at 0° for the angle technique and at 45° and 90° for the two techniques (*p* < 0.05). Contrary to the reference method, the ABC angle method found a statistically significant difference in posterior translation with the knee in extension.

**Conclusion:**

Our technique provides a reliable radiographic measurement of posterior translation with the knee in extension, which should make it easier to acquire radiographs in patients who have pain with knee flexion. This angular measurement also has the advantage of not needing length calibration contrary to the reference technique.

**Level of evidence:**

IV

## Introduction

Severe knee injuries with cruciate ligament tears lead to clinical and radiographical anteroposterior laxity. In cases of isolated posterior cruciate ligament (PCL) tears, radiographic quantification of the posterior translation is essential to determining whether to proceed with conservative or surgical treatment. Currently, the gold standard technique consists of measuring the distance in millimeters of the medial and/or lateral posterior tibial translation with the knee flexed between 70° and 90° during the application of a posterior stress: posterior translation radiographs with manual stress application [1], stress applied with a pulley system [2], Rolimeter [3], TELOS or KT-1000 [4]. The consensus in the literature appears to be that having a difference of at least 8 mm in the posterior translation after applying a posterior stress confirms that the PCL is completely torn [1, 2]. Nevertheless, the reliability and validity of all these techniques requires strict control over tibial rotation, no concurrent anterior cruciate ligament or collateral ligament tears and that patients are able to flex their knee to 90° despite pain associated with post-traumatic hemarthrosis.

The aim of this cadaver study was to evaluate an original technique for measuring posterior tibial translation based on an angle value instead of a distance value with the knee in 0°, 45° and 90° flexion, with and without posterior stress application. It was hypothesized that an angle measurement of the posterior tibial translation would confirm the presence of a PCL tear with the knee flexed and completely extended.

## Method

To carry out this study, ten fresh frozen, non-formalin fixed cadaver knees were screened. These specimens were part of our facility’s scientific donation program. The research protocol was approved by our facility’s ethics committee. We did not have any information about the age of the cadavers. We considered these knees to be independent; however we could not ascertain whether any two knees came from the same subject. The specimens consisted of ten legs, disarticulated at the hip joint. A knee was excluded if it had a visible scar from previous surgery, was clinically unstable or had non-functional ACL or PCL discovered during arthroscopy (visually and based on hook test). Of the ten available knees, seven had not previously undergone surgery and had intact cruciate ligaments. There were four right knees and three left ones.

### Experimental set-up

**Fig. 1:**
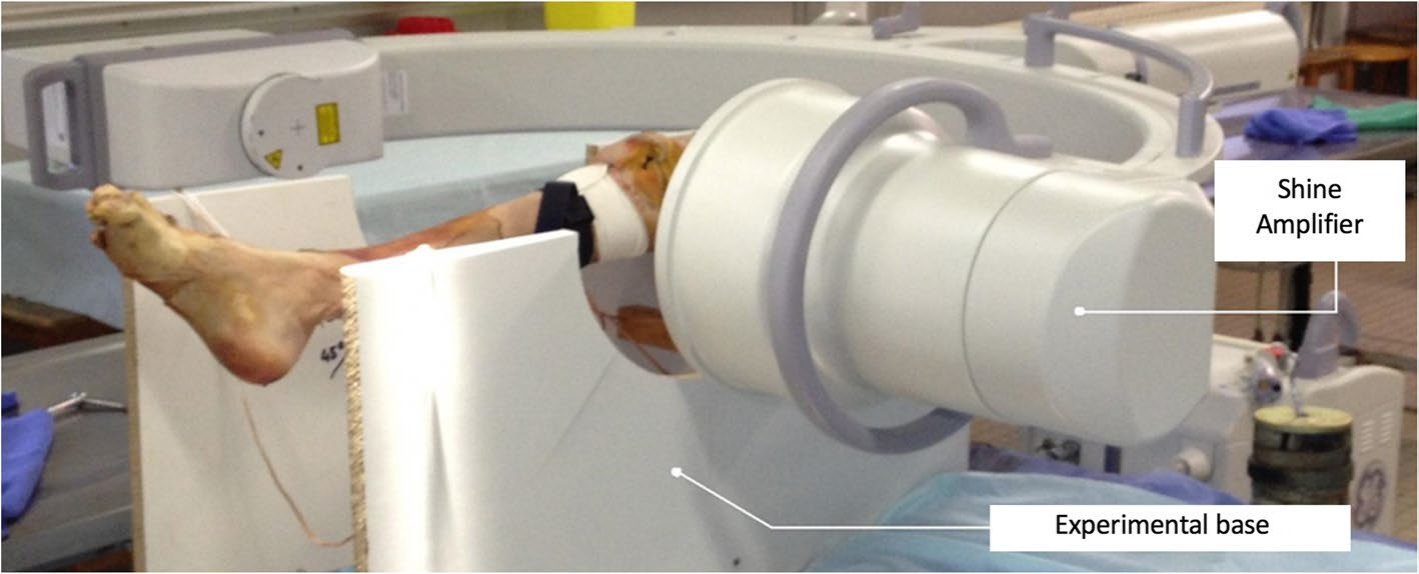
Experimental set-up and lateral radiographic image of the knee in extension with posterior stress

After thawing at room temperature for 48 h, the knees were placed on a wooden base, and the femur secured with two screws placed horizontally, while letting the knee move freely between 0° and 130° flexion but preventing rotational movements (Fig. 1). The knee was placed in the middle of a work area formed by two vertical boards. The opening allowed for reproducible positioning and aligning of the base of the image intensifier (OEC Brivo Plus®, GE Healthcare). An 8 kg weight was hooked on the quadriceps tendon and quadriceps muscle belly to prevent patella infera, while ensuring the tibia could not translate anteriorly or posteriorly on the radiographs.

A posterior stress (PS) was applied by securing a circular strap 10 cm under the tibial tuberosity; the weight was hooked to a suture at the posterior end of the strap. Posterior traction was held perpendicular to the tibial axis using pulleys, independent of the knee flexion angle (0°, 45° or 90°). Posterior traction was controlled by a dynamometer to ensure that a consistent 150 N load was applied.

### Experimental protocol

The main endpoint was the change in posterior translation evaluated by a new method: the ABC angle. This measurement was compared to the conventional posterior translation measurement (distance in millimeters) [5, 6] with and without PS application.

A set of control lateral radiographic images of the knee were made by fluoroscopy with the cadaver knee in 0°, 45° and 90° flexion. These images were taken again after applying a PS of 15 kg. Next, the PCL was transected, and additional images were taken with and without the PS.

To create the PCL injury, a medial parapatellar approach was made with the knee flexed; after confirming that the ACL and PCL were intact, all the femoral attachments of the PCL were cut with a scalpel. We chose to use this precise location for detachment based on our analysis of various anatomical studies; this technique produces a complete, extensive and reproducible transection of the PCL [7, 8]. Before continuing with the experiment, the medial patellar retinaculum was re-sutured to stabilize the patella.

Fluoroscopy images were taken successively in 0°, 45° and 90° flexion in the following sequence:

- Intact knee without PS (IK PS–), intact knee with PS (IK PS +)
- Injured knee without PS (PCL– PS–), injured knee with PS (PCL– PS +)

### Radiographic measurements

The digital images were processed using OSIRIX® 5.8.2 software (Pixmeo) for reading DICOM images. This software was used to measure distances and angles. A 5-cm long pin was inserted vertically in the skin over the patellar tendon to serve as a length calibration guide.

**Fig. 2:**
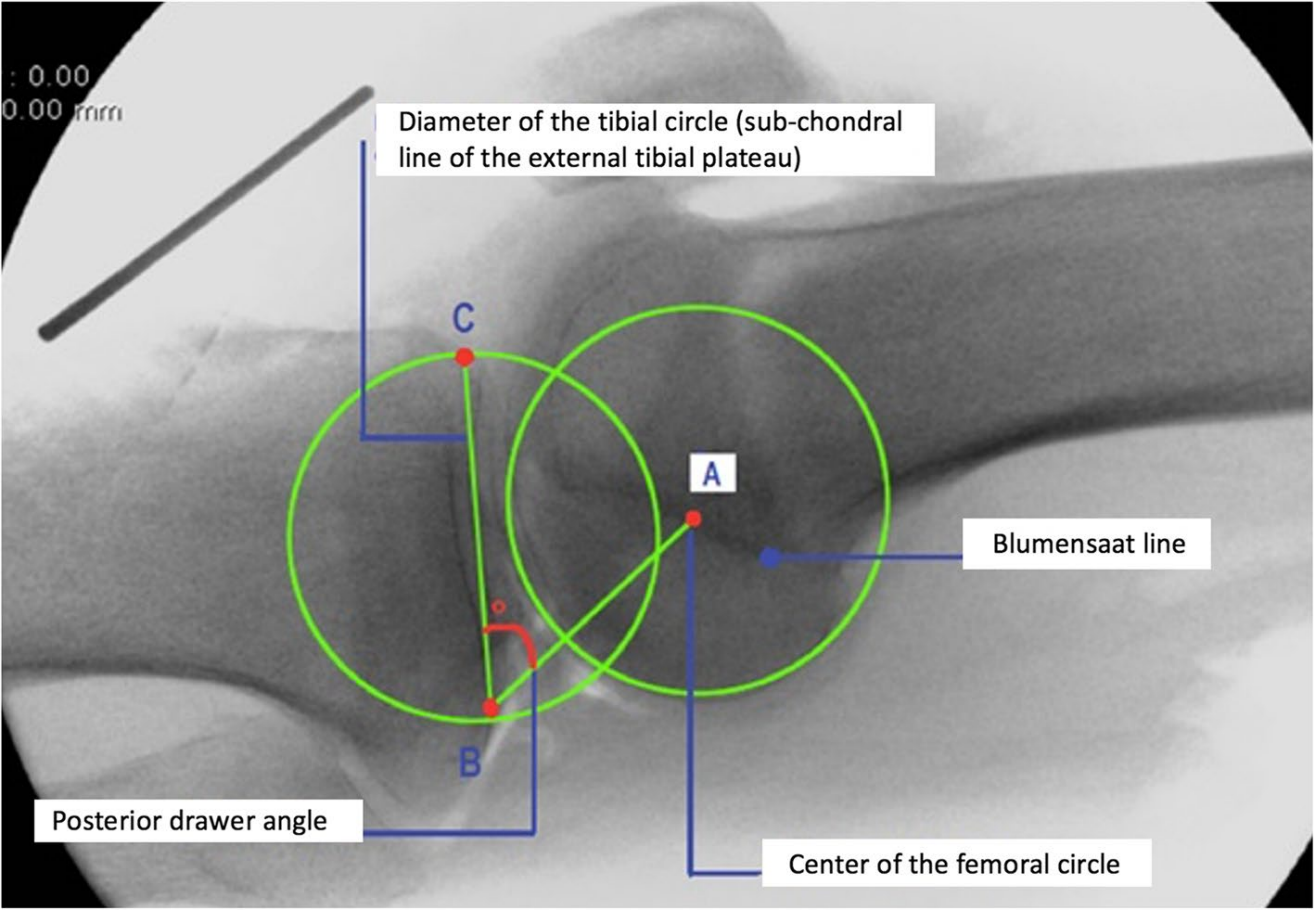
A set of control lateral radiographic images of the knee were made by fluoroscopy with the cadaver knee in 0°, 45° and 90° flexion. Radiographic image of the knee in extension with posterior stress. (Point A: Center of first circle (defining the knee’s center of rotation), Point B: Posterior end of the second circle’s diameter, Point C: Anterior end of the second circle’s diameter)

A circle was drawn at the femoral condyles using the OSIRIX® software. The contours of the circle followed the posterior condyles, by analogy with the circle described by Elias [9], which gave the best approximation of the knee’s center of rotation. This center of rotation always corresponded to a point located on the Blumensaat line (point A). Next, a second circle was drawn on the tibia: the dense subchondral lines merged with the circle’s diameter line and corresponded to the convex shape of the lateral tibial plateau. The ends of the diameter line corresponded to the ends of the tibial circle (points B, C). The center of the circle was the center of the lateral tibial plateau. Next, the ABC angle was measured between the three points using the OSIRIX® software (Fig. 2):

- Point A: Center of first circle (defining the knee’s center of rotation)
- Point B: Posterior end of the second circle’s diameter
- Point C: Anterior end of the second circle’s diameter

Thus, the ABC angle was open to the front and decreased as the posterior translation increased.

**Fig. 3:**
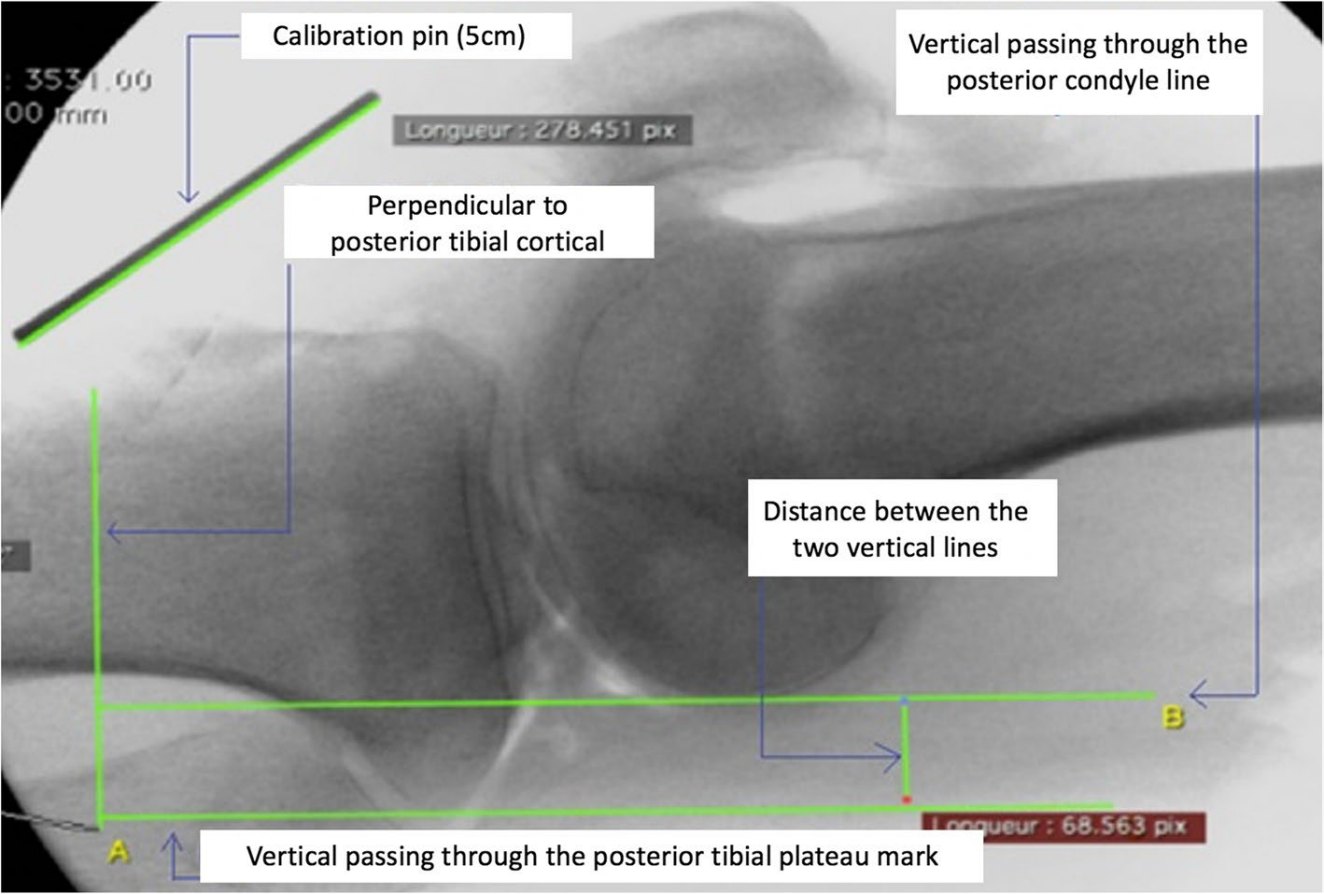
Analysis of a lateral radiographic image of the knee in extension with posterior stress with the conventional translation technique measurement

To compare the ABC angle to the conventional distance measurement of posterior translation, the method described by Jacobsen was used [5, 6]. Two lines perpendicular to a line tangent to the medial tibial plateau were traced, one passing through the posterior edge of the medial tibial plateau and the other adjacent to the posterior edge of the medial condyle (Fig. 3). The gap measured in millimeters was positive when a posterior translation was present and negative when an anterior translation was present.

The two methods for measuring the posterior translation (angle, distance) were performed and validated independently by two experienced orthopedic surgeons.

### Statistical analysis

The statistical analysis was performed with IBM SPSS® Statistics software (version 19.0). Wilcoxon paired tests (non-parametric) were used to compare the distribution of each parameter and analyze the changes in posterior translation in the various experimental conditions. The null hypothesis (H_0_) was that the distributions of the posterior translation values are equal between the two methods. The significance threshold was set at *p* < 0.05. The inter-rater correlation for the angle and distance measurements of posterior tibial translation on the lateral radiographic views was determined with the Kappa correlation coefficient; the inter-rater agreement was expressed as a percentage.

## Results

The posterior translation was measured before and after PCL transection in seven cadaver knees using the experimental protocol described above. After acquiring images in 0°, 45° and 90° flexion, a total of 12 images were analyzed for each of the seven cadaver specimens (Table 1).

**Table 1:** Angular measurement (ABC angle) and conventional distance measurement with and without posterior stress after posterior cruciate ligament section

Posterior translation after PCL rupture	ABC angular measurement (°)						Conventional translation measurement (mm)					
	0° of Flexion		45° of Flexion		90° of Flexion		0° of Flexion		45° of Flexion		90° of Flexion	
	**PS-**	**PS + **	**PS-**	**PS + **	**PS-**	**PS + **	**PS-**	**PS + **	**PS-**	**PS + **	**PS-**	**PS + **
Knee 1	52	49	42	39	40	39	2,5	4,8	2,5	6,0	9,7	14,4
Knee 2	52	50	57	44	56	56	12,1	13,5	3,3	11,2	10,4	13,9
Knee 3	54	50	49	46	55	46	3,8	3,5	1,7	2,7	4,7	9,3
Knee 4	51	50	50	48	48	48	7,3	6,5	5,0	5,7	6,7	4,7
Knee 5	59	55	47	45	52	45	2,5	3,3	8,2	8,8	9,2	15,2
Knee 6	65	59	46	46	56	51	-8,3	-1,3	3,0	4,0	7,3	12,8
Knee 7	54	51	50	41	51	40	0,0	3,9	5,3	8,7	7,5	12
Mean	55,29	52,00	48,71	44,14	51,14	46,43	2,8	4,9	4,1	6,7	7,9	11,8
Median	54	50	49	45	52	46	2,5	3,9	3,3	6,0	7,5	12,8
Standard deviation	5,02	3,65	4,61	3,13	5,73	5,97	6,31	4,49	2,20	2,99	1,97	3,66

Application of a PS revealed clear changes in the posterior translation after PCL transection with the knee at 0° for the angle technique only (*p* < 0.05). Contrary to the conventional distance measurement with PS, the ABC angle method found a statistically significant difference in the posterior translation with the knee in extension (Table 2).

**Table 2:** Posterior translation variation analysis by non parametric test (Wilcoxon)

**Posterior translation comparison**		**0° (mm)**	**0° (Degrees)**	**45° (mm)**	**45° (Degrees)**	**90° (mm)**	**90° (Degrees)**
**Lesion type**	**PCL + PS + / PCL-PS + **	NS	**0,028**	0,043	0,017	0,028	0,018
**Effect of posterior stress**	**PCL-PS- / PCL-PS + **	NS	0,018	0,018	0,026	0,028	0,026
	**PCL + PS- / PCL + PS + **	NS	NS	NS	NS	NS	NS

*PCL* Posterior cruciate ligament, *PS* Posterior stress

In 45° and 90° flexion, both measurement methods found a posterior translation after PCL transection (*p* < 0.05). The inter-rater agreement was 98% for the entire measurement series, with a Kappa coefficient of 0.96 [0.88–1.0].

## Discussion

The most important finding of the present study was that the ABC angle method identified a significant change in posterior translation when the knees were in full extension. However, applying a posterior stress produced a significant posterior translation in cadaver knees with a transected PCL in 45° and 90° flexion that was measured with the two methods.

In the literature, only a few studies have reported the posterior translation with the knee in full extension with PS after PCL injury and/or found no significant difference in this position when using the distance measurement method [6, 10]. Also, disinterest in analyzing posterior tibial translation in extension is due to several biomechanical studies, including those by Li [11], Pearsall [12] and Castle et al. [13], reporting a significant increase in the measured values of posterior translation while the knee was being flexed to 90°. In our study, 45° and 90° of flexion were the best positions for revealing changes in posterior translation. The mean of posterior translation after PCL transection when PS was applied was 4.7 mm in 0° while it was 11.8 mm in 90°. Several anatomical studies have described the two bundles of the PCL. We know that anterolateral bundle is taut during flexion and posteromedial bundle taut during extension and also 90° of flexion [1, 12, 14]. The extension testing makes it possible to detect an injury of the posteromedial bundle in case of partial rupture.

The ABC angle method for measuring posterior translation makes it possible to detect and/or diagnose PCL tears with better sensitivity during a measurement with the knee extended; the findings are generally negative when using the conventional distance measurement method. Also, using the ABC angle method is a radiographic alternative to the distance measurement method described by Jacobsen [5, 6], and facilitates the acquisition of radiographs in a patient who has pain during knee flexion. This angular measurement also has the advantage of not needing length calibration contrary to the reference technique. In fact, an angular measurement is free from any calibration, which makes this technique more easily reproducible.

During our experiment, the PS consisted of a circular strap fixed 10 cm below the tibial tubercle; the posterior portion of the strap had a suture on which 15 kg of load was attached; this means that 150 N load was applied to each specimen. This load was chosen by analogy with various stress radiography studies and was comparable to the load applied with the TELOS® that Badet [15] used during post-PCL reconstruction studies. Other laximetry techniques have been described such as the Rolimeter or the KT-1000 arthrometer; however, these are only moderately useful for measuring posterior laxity in knees with a PCL tear [4].

The angle measurement described here uses landmarks that are not affected by movement of the distal femoral epiphysis during knee flexion and extension; instead, it uses a landmark located in the knee’s center of rotation (point A). Badet [15] suggested using this type of central landmark because it would better capture the tibiofemoral translation. Despite devices being available to stabilize the tibia and femur, application of a PS reduces the tibial epiphyseal flexion, which is only considered in our angle measurement method.

Also note that our experimental protocol was focused solely on sagittal posterior translation. Some biomechanical studies, such as the one by Li et al. [11] found external rotation beyond 60° flexion, which was not factored into our analysis.

The cadaver specimens were left out at room temperature for 48 h to thaw them before the experiment. We sought to combine the optimal conditions for measuring knee laxity by reproducing the elasticity/solidity relationship that is as close as possible to clinical practice on a living patient, while getting around any bias associated with reflex contractions. Nevertheless, performing this study on fresh cadaver knees would allow us to come even closer to reproducing the knee laxity measurement conditions in living patients.

The limitations of this study are those of a cadaveric study with a small sample size, and without initial power analysis. A prospective study with a larger sample size would be useful for defining a threshold value for detecting the presence of a PCL tear.

## Conclusion

The ABC angle measurement method described here was able to detect significant sagittal posterior translation when PS was applied in cadaver knees with isolated PCL transection in 0°, 45° and 90° flexion. Unlike the conventional distance measurement method, this new technique found a statistically significant difference with the knee extended and may be a reliable alternative to the gold standard method described by Jacobsen [5, 6], while facilitating the acquisition of radiographs in patients who have pain during knee flexion. This angular measurement also has the advantage of not needing length calibration contrary to the reference technique.

## References

[1] Kumagai M, Mizuno Y, Mattessich SM, Elias JJ, Cosgarea AJ, Chao EY (2002) Posterior cruciate ligament rupture alters in vitro knee kinematics. Clin Orthop 241–248. 10.1097/00003086-200202000-00029 10.1097/00003086-200202000-00029 11937888

[2] Jacobsen K (1976). Stress radiographical measurement of the anteroposterior, medial and lateral stability of the knee joint. Acta Orthop Scand.

[3] Jacobsen K (1981). Gonylaxometry. Stress radiographic measurement of passive stability in the knee joints of normal subjects and patients with ligament injuries. Accuracy and range of application. Acta Orthop Scand Suppl.

[4] Makris CA, Georgoulis AD, Papageorgiou CD, Moebius UG, Soucacos PN (2000). Posterior cruciate ligament architecture: evaluation under microsurgical dissection. Arthroscopy.

[5] Edwards A, Bull AMJ, Amis AA (2007). The attachments of the fiber bundles of the posterior cruciate ligament: an anatomic study. Arthroscopy.

[6] Elias SG, Freeman MA, Gokcay EI (1990) A correlative study of the geometry and anatomy of the distal femur. Clin Orthop Relat Res 260:98–103 2225651

[7] Höher J, Akoto R, Helm P, Shafizadeh S, Bouillon B, Balke M (2015). Rolimeter measurements are suitable as substitutes to stress radiographs in the evaluation of posterior knee laxity. Knee Surg Sports Traumatol Arthrosc.

[8] Castle TH, Noyes FR, Grood ES (1992) Posterior tibial subluxation of the posterior cruciate-deficient knee. Clin Orthop Relat Res 284:193–202 1395293

[9] Huber FE, Irrgang JJ, Harner C, Lephart S (1997). Intratester and intertester reliability of the KT-1000 arthrometer in the assessment of posterior laxity of the knee. Am J Sports Med.

[10] Li G, Gill TJ, DeFrate LE, Zayontz S, Glatt V, Zarins B (2002). Biomechanical consequences of PCL deficiency in the knee under simulated muscle loads–an in vitro experimental study. J Orthop Res.

[11] Pearsall AW, Hollis JM (2004). The effect of posterior cruciate ligament injury and reconstruction on meniscal strain. Am J Sports Med.

[12] Grassmayr MJ, Parker DA, Coolican MRJ, Vanwanseele B (2008). Posterior cruciate ligament deficiency: biomechanical and biological consequences and the outcomes of conservative treatment. A systematic review. J Sci Med Sport.

[13] Hewett TE, Noyes FR, Lee MD (1997). Diagnosis of complete and partial posterior cruciate ligament ruptures. Stress radiography compared with KT-1000 arthrometer and posterior drawer testing. Am J Sports Med.

[14] Bowman KF, Sekiya JK (2010). Anatomy and biomechanics of the posterior cruciate ligament, medial and lateral sides of the knee. Sports Med Arthrosc Rev.

[15] Kowalczuk M, Leblanc MC, Rothrauff BB, Debski RE, Musahl V, Simunovic N, Ayeni OR (2015). Posterior tibial translation resulting from the posterior drawer manoeuver in cadaveric knee specimens: a systematic review. Knee Surg Sports Traumatol Arthrosc.

